# Tumor necroptosis promotes metastasis through modulating the interplay between tumor and host immunity

**DOI:** 10.18632/oncotarget.28404

**Published:** 2023-04-10

**Authors:** Zhaoshan Liu, Swati Choksi, Zheng-Gang Liu

**Keywords:** necroptosis, breast cancer, metastasis, E-cadherin, immunity

Cell death could happen as finely regulated programmed cell death (PCD) or non-regulated accidental cell death (necrosis). In addition to apoptosis, which is the first known PCD, several other forms of PCD such as necroptosis and ferroptosis have been identified and the underlying mechanisms of these forms of cell death have been well studied [1]. For necroptosis, a regulated necrotic cell death, Mixed lineage kinase domain-like protein (MLKL) was found to be the key executor of the death process and its oligomerization and translocation to the plasma membrane results in the shedding of cell surface proteins and the rupture of the cell plasma membrane of necroptotic cells [2, 3]. The role of the different forms of PCD in tumorigenesis has been extensively investigated. While apoptosis and ferroptosis inhibit tumor progression, necroptosis seems to have a promoting role in tumor progression [4]. We recently found that necroptosis is the main form of cell death observed in tumor necrosis, the foci of cell death in core regions of solid tumors under hypoxia and nutrient deprivation [5]. More importantly, we found that blocking necroptosis leads to the inhibition of metastasis in mouse breast cancer models [5–7].

Tumor metastasis is the main cause of cancer mortality and is a complex process modulated by many different factors including the interplay between tumor cells and host immunity. As host T cells play a critical role in controlling metastasis by exerting their anti-tumor activity as tumor-infiltrating T cells and peripheral T cells, inhibiting the anti-tumor activity of T cells is a major feature of the interplay of tumor cells and host immunity during tumor development and metastasis. While recent studies reported that necroptosis may play an important role in tumorigenesis, the exact role of necroptosis in tumor development may need to be further evaluated in different types of tumors. In two preclinical mouse breast cancer models, a genetically modified MMTV-PyMT model and an orthotopic transplantation MVT-1 model, we found that blocking tumor necroptosis by MLKL deletion resulted in the dramatic reduction of lung metastasis and the increase of the anti-tumor activity of tumor infiltrating and peripheral blood T cells [6]. Importantly, the data suggests that necroptosis has a suppressive effect on anti-tumor activity of CD8+ T cells and this inhibition of peripheral T cells may promote the survival of circulating tumor cells.

We previously found that the translocation of MLKL leads to the activation of the cell-surface proteases, A Disintegrin And Metalloproteases (ADAMs), which causes ectodomain shedding of many cell surface proteins from necroptotic cells [3]. As several of the shedded cell surface proteins, E-cadherin, JAM-A, syndecan-1, are implied in promoting metastasis, we examined the levels of these proteins and found that their levels are dramatically reduced in MLKL null tumors/mice. Importantly, we showed that the administration of the ADAM10/ADAM17 inhibitor GW280264X in mice with WT tumors reduces the levels of these soluble proteins and leads to the increased anti-tumor activity of T cells and the dramatic decrease of metastasis. This data suggests that the ectodomain shedding of cell surface proteins of necroptotic cells promotes tumor metastasis through inhibiting the anti-tumor activity of T cells.

As soluble E-cadherin (sE-cadherin) is a ligand for Killer cell lectin-like receptor subfamily G member 1 (KLRG1), a known co-inhibitory receptor of T cell, we showed that sE-cadherin inhibits T cell anti-tumor activities through KLRG1 and that neutralizing KLRG1 with an anti-KLRG1 antibody dramatically reduces lung metastasis comparable to that seen when necroptosis is blocked with MLKL deletion.

**Figure 1:**
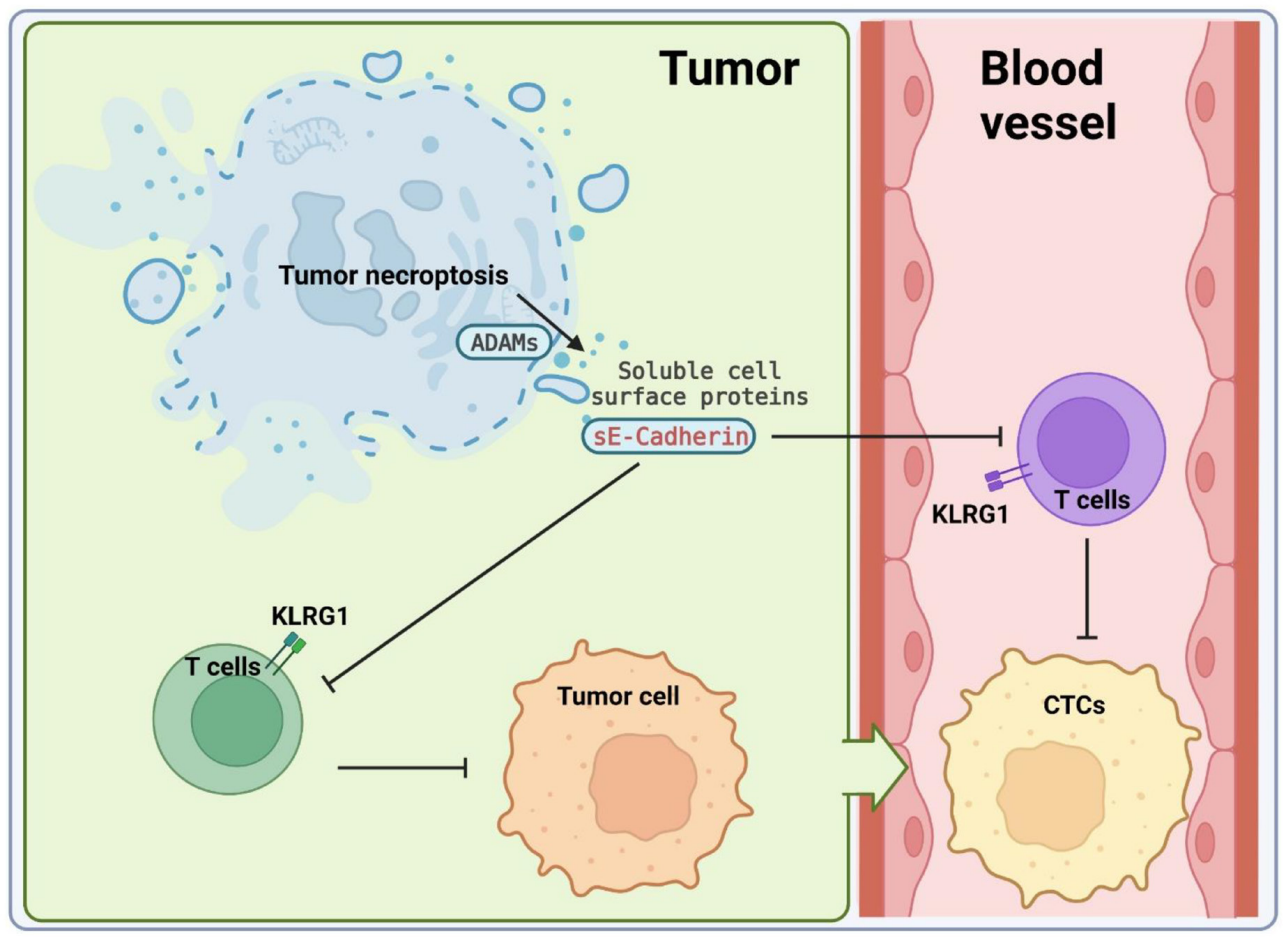
Tumor necroptosis promotes metastasis of breast cancer by suppressing anti-tumor immunity. Necroptosis leads to the activation ADAMs, which causes ectodomain shedding of cell surface proteins. Soluble E-cadherin binds to KLRG1 inhibiting anti-tumor activity of T cells and this inhibition of T cells may promote the survival of circulating tumor cells (CTCs).

In summary, our study suggests that necroptosis-mediated shedding of the surface proteins of tumor cells play a key role in inhibiting the anti-tumor activity of T cells and promoting metastasis (Figure 1). These findings also provide compelling evidence for targeting ADAM proteases or necroptosis as a promising therapy in controlling metastasis of breast cancer. The small-molecule inhibitors of ADAMs have been explored as potential cancer therapeutics. Some of these inhibitors such as INCB7839, GI254023X, GW280264X, LT4, and MN8 have been tried with considerable success against diverse cancers. To pharmaceutically block necroptosis, multiple small molecules that target Receptor Interacting Protein Kinase 1 and 3 (RIPK1 and RIPK3) have been developed. However, due to the toxicity of these drugs and that RIPK1 may not be involved in necroptosis in tumor, applications of these inhibitors of RIPK1 and RIPK3 in cancer treatment have not been successful [4, 7]. Developing novel necroptosis inhibitor by targeting other proteins such as MLKL may be critical for successfully blocking necroptosis in cancer and mitigating metastasis.
